# $\Omega _c$ excited states within a $\mathrm{SU(6)}_{\mathrm{lsf}}\times$ HQSS model

**DOI:** 10.1140/epjc/s10052-018-5597-3

**Published:** 2018-02-07

**Authors:** J. Nieves, R. Pavao, L. Tolos

**Affiliations:** 1 Instituto de Física Corpuscular (centro mixto CSIC-UV), Institutos de Investigación de Paterna, Aptdo. 22085, 46071 Valencia, Spain; 2 Institut für Theoretische Physik, University of Frankfurt, Max-von-Laue-Str. 1, 60438 Frankfurt am Main, Germany; 3 Frankfurt Institute for Advanced Studies, University of Frankfurt, Ruth-Moufang-Str. 1, 60438 Frankfurt am Main, Germany; 4 Institute of Space Sciences (ICE, CSIC), Campus UAB, Carrer de Can Magrans, 08193 Barcelona, Spain; 5 Institut d’Estudis Espacials de Catalunya (IEEC), 08034 Barcelona, Spain

## Abstract

We have reviewed the renormalization procedure used in the unitarized coupled-channel model of Romanets et al. (Phys Rev D 85:114032, 2012), and its impact in the $C=1$, $S=-\,2$, and $I=0$ sector, where five $\Omega _c^{(*)}$ states have been recently observed by the LHCb Collaboration. The meson-baryon interactions used in the model are consistent with both chiral and heavy-quark spin symmetries, and lead to a successful description of the observed lowest-lying odd parity resonances $\Lambda _c(2595)$ and $\Lambda _c(2625)$, and $\Lambda _b(5912)$ and $\Lambda _b(5920)$ resonances. We show that some (probably at least three) of the states observed by LHCb will also have odd parity and $J=1/2$ or $J=3/2$, belonging two of them to the same $\mathrm{SU(6)}_{\mathrm{light}\text {-}\mathrm{spin}\text {-}\mathrm{flavor}}\times$ HQSS multiplets as the latter charmed and beauty $\Lambda$ baryons.

## Introduction

The LHCb Collaboration [1] has recently reported the existence of five $\Omega _c$ states, analyzing the $\Xi _c^+ K^-$ spectrum in *pp* collisions, with masses ranging between 3 and 3.1 GeV. These results have renewed the interest in baryon spectroscopy, with the long-standing question whether these states can be accommodated within the quark model picture and/or qualify better as being dynamically generated via hadron-hadron scattering processes.

Earlier predictions for such states have been reported within conventional quark models [2–13]. The experimental discovery of the five $\Omega _c$ states has triggered a large activity in the field, and thus some quark models have been revisited in view of the new results [14–20], suggestions as pentaquarks have been advocated [21–25], models based on QCD sum-rules have been put to test [26–32], or quark-soliton models have been employed [33]. Also, Lattice QCD has reported results on the spectroscopy of $\Omega _c$ states [34].

Within molecular models, there have been previous predictions on $\Omega _c$ states [35–38]. In Ref. [36] several resonant states were obtained with masses much below 3 GeV, by employing a zero-range exchange of vector mesons as the bare interaction for the *s*-wave baryon-meson scattering. Similar qualitative results were obtained in Ref. [35], where finite range effects were considered. Lately the work of Ref. [39] has revisited Ref. [36], finding that, after modifying the regularization scheme with physically motivated parameters, two $\Omega _c$ resonant states were generated at 3050 and 3090 MeV with spin-parity $J^P=1/2^-$, reproducing the masses and widths of two of the experimental states. More recently, the $\Omega _c$ states have been also investigated using an extended local hidden gauge approach [40]. Within this scheme, low-lying $1/2^+$ and $3/2^+$ baryons, as well as pseudoscalar and vector mesons, are considered to construct the baryon-meson coupled channel space. In this manner, two $\Omega _c$ states of $J^P=1/2^-$ and one $\Omega _c^*\;J^P=3/2^-$ can be identified, the first two in good agreement with the results of [39] and the third one fairly well.

The use of the hidden-gauge formalism allows for the preservation of heavy-quark spin symmetry (HQSS), which is a proper QCD symmetry that appears when the quark masses, such as that of the charm quark, become larger than the typical confinement scale. Aiming to incorporate explicitly HQSS, a scheme was developed in Refs. [37, 38, 41–43] that implements a consistent $\mathrm{SU(6)}_{\mathrm{lsf}} \times \, \mathrm{SU(2)}_\mathrm{HQSS}$ extension of the Weinberg–Tomozawa (WT) $\pi N$ interaction, where “lsf” stands for light quark-spin-flavor symmetry, respectively. Indeed, the works of Refs. [37, 38] are the first meson-baryon molecular studies, fully consistent with HQSS, of the well-established odd-parity $\Lambda _c(2595)$ [$J=1/2$] and $\Lambda _c(2625)$ [$J=3/2$] resonances.

Within this scheme in the $J=1/2$ sector, one finds a pole structure that mimics the well-known two-pole pattern of the $\Lambda (1405)$ [44–48]. Thus, in the region of 2595 MeV, two states are dynamically generated. The first one, identified with the $\Lambda _c(2595)$ resonance, is narrow and strongly couples to the *ND* and $ND^*$ channels, with a negligible coupling to the open $\Sigma _c\pi$ channel. The second state is quite broad and it has a sizable coupling to this latter channel. On the other hand, the $J^P=(3/2)^-$ state is generated mainly by the $(ND^*, \Sigma _c^*\pi )$ coupled-channel dynamics, and it would be the charm counterpart of the $\Lambda (1520)$. Similar results are also obtained in the extension of the local hidden gauge approach of Ref. [49]. The same scheme also dynamically generates the $\Lambda _b(5912)$ and $\Lambda _b(5920)$ narrow resonances, discovered by LHCb in 2012 [50], which turn out to be HQSS partners, naturally explaining in this way their approximate mass degeneracy [42]. Moreover, the $\Lambda _b(5920)$ resonance turns out to be the bottom version of the $\Lambda _c(2625)$ one, while the $\Lambda _b(5912)$ would not be the counterpart of the $\Lambda _c(2595)$ resonance, but it would be of the second charmed state that appears around 2595 MeV, and that gives rise to the two-pole structure mentioned above [42].

**Table 1:** $\Omega _c$ an $\Omega _c^*$ states, reported in Ref. [38], coming from the most attractive $\mathrm{SU(6)}_{\mathrm{lsf}} \times$ HQSS representations. We label those states from **a** to **e**, according to their position in energy

Name	$M_R$ (MeV)	$\Gamma _R$ (MeV)	*J*
**a**	2810.9	0	1/2
**b**	2814.3	0	3/2
**c**	2884.5	0	1/2
**d**	2941.6	0	1/2
**e**	2980.0	0	3/2

In Ref. [38] five $\Omega _c$ states were found, three $J=1/2$ and the two $J=3/2$ bound states, the positions being shown in Table VI of that reference or in Table 1 in the present work. These states come from the most attractive $\mathrm{SU(6)}_{\mathrm{lsf}}\times$ HQSS representations. Attending to the breaking pattern of the spin-flavor SU(8) symmetry discussed in Ref. [38], the two lowest-lying $\Omega _c$ and $\Omega ^*_c$ states (**a** and **b**) and the $\Lambda _c(2595)$ would be members of the same **21** $\mathrm{SU(6)}_{\mathrm{lsf}}$ multiplet, while both, the third $\Omega _c$ (**c**) and the $\Lambda _c(2625)$ resonances would be in the **15** $\mathrm{SU(6)}_{\mathrm{lsf}}-$ irreducible representation. Finally, the two heaviest $\Omega _c$ and $\Omega ^*_c$ states (**d** and **e**) reported in [38] would not be directly related to the $\Lambda _c(2595)$ and $\Lambda _c(2625)$ resonances, since they would stem originally from a different SU(8) representation. These five odd-parity $\Omega _c, \Omega ^*_c$ states, coming from the most attractive $\mathrm{SU(6)}_{\mathrm{lsf}}\times$  HQSS representations, have masses below 2.98 GeV, and cannot be easily identified with any of the LHCb resonances, located all of them above 3 GeV. Predicted masses, however, depend not only on the baryon-meson interactions, but also on the adopted renormalization scheme (RS). In this work we review the RS used in [38], and its impact in the generation of the $\Omega _c^{(*)}$ states. We show how the pole positions can be moved up by implementing a different RS, making then feasible the identification of at least three states with the observed $\Omega _c^{(*)}$ states by LHCb.

The paper is organized as follows. In Sect. 2 we present the $\mathrm{SU(6)}_{\mathrm{lsf}} \times \mathrm{SU(2)}_\mathrm{HQSS}$ extension of the WT interaction, while in Sect. 3 we show our results for the $\Omega _c^{(*)}$ states and the possible identification of three of them with the experimental ones. Finally, in Sect. 4 we present our conclusions.

## Formalism

We will consider the sector with charm $C=1$, strangeness $S=-\,2$ and isospin $I=0$ quantum numbers, where the $\Omega _c^{(*)}$ excited states are located by revising the results in Ref. [38].

The building-blocks in the $C=1$ sector are the pseudoscalar ($D_s, D, K, \pi ,\eta , {{\bar{K}}}, {{\bar{D}}}, {{\bar{D}}}_s$) and vector ($D_s^*, D^*,K^*, \rho ,\omega , {{\bar{K}}}^{*}, {{\bar{D}}}^*, {{\bar{D}}}_s^*, \phi$) mesons, the spin–1 / 2 octet and the spin–3 / 2 decuplet of low-lying light baryons, in addition to the spin-1/2 ($\Lambda _c$, $\Sigma _c$, $\Xi _c$, $\Xi '_c$, $\Omega _c$), and spin-3/2 ($\Sigma ^*_c$, $\Xi ^*_c$, $\Omega _c^*$) charmed baryons [38, 43]. All baryon-meson pairs with $(C=1, S=-\,2, I=0)$ quantum numbers span the coupled-channel space for a given total angular momentum (*J*). The *s*-wave tree level amplitudes between two channels are given by the $\mathrm{SU(6)}_{\mathrm{lsf}}$ $\times$ HQSS WT kernel

1 $$\begin{aligned} V_{ij}^J(s) = D_{ij}^J \frac{2 \sqrt{s}-M_i-M_j}{4 f_i f_j} \sqrt{\frac{E_i+M_i}{2 M_i}} \sqrt{\frac{E_j+M_j}{2M_j}}, \end{aligned}$$

with $M_i$ and $m_i$, the masses of the baryon and meson in the *i* channel, respectively, and $E_i$ the center-of-mass energy of the baryon in the same channel,

2 $$\begin{aligned} E_i=\frac{s-m_i^2+M_i^2}{2 \sqrt{s}}. \end{aligned}$$

The hadron masses and meson decay constants, $f_i$, have been taken from Ref. [38]. The $D_{ij}^J$ matrices are determined by the underlying $\mathrm{SU(6)}_{\mathrm{lsf}} \times$ HQSS group structure of the interaction. Tables for all of them can be found in the Appendix B of Ref. [38].

We use the matrix $V_{ij}^J$ as potential to solve the Bethe–Salpeter equation (BSE), which leads to a *T*-matrix of the form

3 $$\begin{aligned} T^J(s)=\frac{1}{1-V^J(s) G^J(s)} V^J(s), \end{aligned}$$

satisfying exact unitarity in coupled channels. In the above equation, $G^J(s)$ is a diagonal matrix that contains the loop functions corresponding to the particles of the different channels being considered.

The two-body loop function is given by

4 $$\begin{aligned} G_i(s)=i 2M_i \int \frac{d^4 q}{(2 \pi )^4} \frac{1}{q^2-m_i^2+i\epsilon } \frac{1}{(P-q)^2-M_i^2+i\epsilon }, \end{aligned}$$

with *P* the total momentum of the system such that $P^2=s$. We omit the index *J* from here on for simplicity. The bare loop function is logarithmically ultraviolet (UV) divergent and needs to be renormalized. This can be done by one-subtraction

5 $$\begin{aligned} G_i(s)=\overline{G}_i(s)+G_i(s_{i+}) , \end{aligned}$$

with the finite part of the loop function, $\overline{G}_i(s)$, given in Ref. [51],

6 $$\begin{aligned} \overline{G}_i(s) = \frac{2 M_i}{(4 \pi )^2} \left( \left[ \frac{M_i^2-m_i^2}{s}-\frac{M_i-m_i}{M_i+m_i}\right] \log \frac{M_i}{m_i}+L_i(s) \right) , \end{aligned}$$

where

7a $$\begin{aligned} s_{i-}&=(m_i-M_i)^2, \end{aligned}$$

7b $$\begin{aligned} s_{i+}&=(m_i+M_i)^2, \end{aligned}$$

and for real *s* and above threshold, $s > s_{i+}$

8 $$\begin{aligned} L_i(s+i\epsilon ) = \frac{\lambda ^{\frac{1}{2}}(s, m_i^2,M_i^2)}{s} \left( \log \left[ \frac{1+\sqrt{\frac{s-s_{i+}}{s-s_{i-}}}}{1-\sqrt{\frac{s-s_{i+}}{s-s_{i-}}}} \right] - i \pi \right) , \end{aligned}$$

and $\lambda (x,y,z)$ the ordinary Källen function.

The divergent contribution of the loop function, $G_i(s_{i+})$ in Eq. (5) needs to be renormalized. We will examine here two different renormalization schemes, widely used in the literature.

On the one hand, we will perform one subtraction at certain scale $\sqrt{s}=\mu$, such that

9 $$\begin{aligned} G_i(\sqrt{s}=\mu ) = 0\,. \end{aligned}$$

In this way,

10 $$\begin{aligned} G_i^\mu (s_{i+}) = -\,\, \overline{G}_i(\mu ^2). \end{aligned}$$

so that

11 $$\begin{aligned} G_i^\mu (s) =\overline{G}_i(s) - \overline{G}_i(\mu ^2). \end{aligned}$$

In addition, we use the prescription adopted in Ref. [38], where $\mu$ is chosen to be independent of the total angular momentum *J*, common for all channels in a given *CSI* sector, and equal to

12 $$\begin{aligned} \mu = \sqrt{\alpha \left( m_{th}^2+M_{th}^2 \right) } , \end{aligned}$$

with $m_{th}$ and $M_{th}$ the masses of the meson and baryon of the channel with the lowest threshold in the given *CSI* sector [36, 52], and $\alpha$ a parameter that can be adjusted to data [37]. In what follows, we will refer to this scheme as $\mu -RS$.

In the second RS, we make finite the UV divergent part of the loop function using a sharp-cutoff regulator $\Lambda$ in momentum space, which leads to [53]

13 $$\begin{aligned} G_i^\Lambda (s_{i+})= & {} \frac{1}{4\pi ^2} \frac{M_i}{m_i+M_i} \left( m_i\ln \frac{m_i}{\Lambda + \sqrt{\Lambda ^2+m_i^2}}\right. \nonumber \\&+\, \left. M_i\ln \frac{M_i}{\Lambda + \sqrt{\Lambda ^2+M_i^2}} \right) , \end{aligned}$$

and thus, for the UV cutoff case we have

14 $$\begin{aligned} G^{\Lambda }_i(s) =\overline{G}_i(s) + G_i^{\Lambda }(s_{i+}). \end{aligned}$$

Note that, there are no cutoff effects in the finite $\overline{G}_i(s)-$loop function, as it would happen if the two-body propagator of Eq. (6) would have been directly calculated using the UV cutoff $\Lambda$.

If a common UV cutoff is employed for all channels within a given *CSI* sector, both RSs are independent and will lead to different results. However, if one allows the freedom of using channel-dependent cutoffs, the one-subtraction RS, $\mu -RS$, is recovered by choosing in each channel, $\Lambda _i$ such that

15 $$\begin{aligned} G^{\Lambda _i}_i(s_{i+})= -\overline{G}_i(\mu ^2) . \end{aligned}$$

The dynamically-generated $\Omega _c$ resonances can be obtained as poles of the scattering amplitudes in each *J* sector for $(C=1,S=-\,2, I= 0)$. We look at both the first and second Riemann sheets (FRS and SRS) of the variable $\sqrt{s}$. The poles of the scattering amplitude on the FRS that appear on the real axis below threshold are interpreted as bound states. The poles that are found on the SRS below the real axis and above threshold are identified with resonances.^1^ The mass and the width of the bound state/resonance can be found from the position of the pole on the complex energy plane. Close to the pole, the *T*-matrix behaves as

16 $$\begin{aligned} T_{ij}(s) \simeq \frac{g_i g_j}{\sqrt{s}-\sqrt{s_R}}. \end{aligned}$$

The quantity $\sqrt{s_R}=M_R - \mathrm {i}\, \Gamma _R/2$ provides the mass ($M_R$) and the width ($\Gamma _R$) of the state, and $g_i$ is the complex coupling of the resonance to the channel *i*.

The couplings $g_i$ are obtained by first assigning an arbitrary sign to one of them, say $g_1$. Then, we have that

17 $$\begin{aligned} g_1^2=\lim _{\sqrt{s}\rightarrow \sqrt{s_R}} (\sqrt{s}-\sqrt{s_R})T_{11}(s) , \end{aligned}$$

and the other couplings result from

18 $$\begin{aligned} g_j = g_1 \lim _{\sqrt{s}\rightarrow \sqrt{s_R}} \frac{T_{1j}(s)}{T_{11}(s)} . \end{aligned}$$

In order to analyze the contribution of each baryon-meson channel to the generation of a resonance, one has to not only analyze the coupling but also the size of each baryon-meson loop, since the product $g_i G_i(s_R)$ gives the strength of the wave function at the origin for *s*-wave [54].

## Results

The LHCb experiment has analyzed the $\Xi _c^+ K^-$ spectrum using *pp* collisions and five new narrow excited $\Omega _c^0$ states have been observed: the $\Omega _c^0(3000)$, $\Omega _c^0(3050)$, $\Omega _c^0(3066)$, $\Omega _c^0(3090)$ and the $\Omega _c^0(3119)$, the last three also seen in the $\Xi _c^{'+} K^-$ decay. Moreover, a sixth broad structure around 3188 has also been found in the $\Xi _c^+ K^-$ spectrum.

**Fig. 1:**
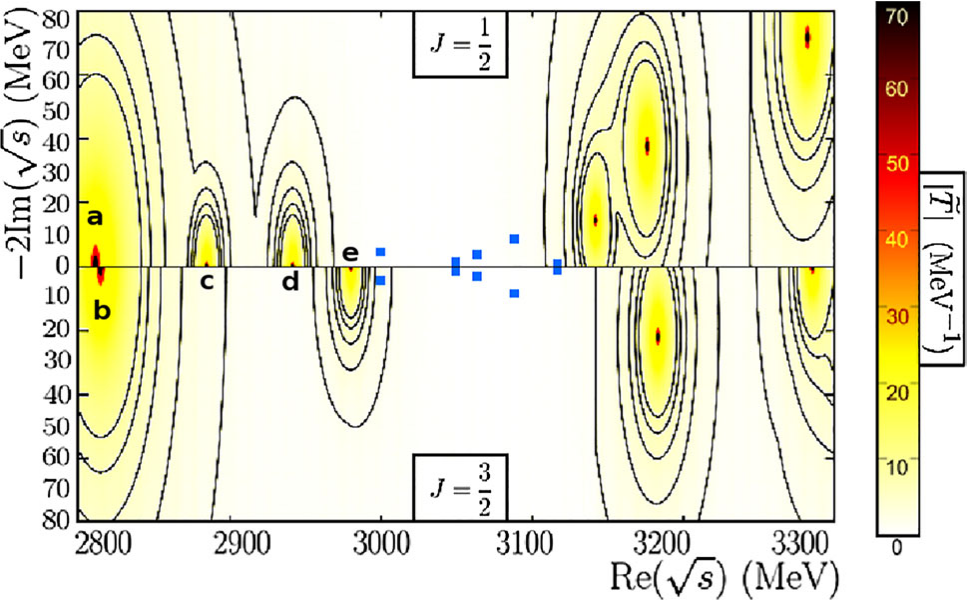
$\Omega _c (J=1/2)$ an $\Omega _c^* (J=3/2)$ odd-parity states, reported in Ref. [38], coming from the most attractive $\mathrm{SU(6)}_{\mathrm{lsf}} \times$ HQSS representations. These five states, denoted as in Table 1, are located below 3 GeV for $J=1/2$ (upper plot) and $J=3/2$ (lower plot), while the five heavier resonant states above 3 GeV, also shown, come from less attractive $\mathrm{SU(6)}_{\mathrm{lsf}} \,\times \,$ HQSS multiplets, stemming from the exotic **4752** SU(8) representation. Since the dynamically generated states may couple differently to their baryon-meson components, we show the $ij-$channel independent quantity $|{\tilde{T}}(z)|_J = \mathrm{max}_j \sum _i |T_{ij}^J(z)|$, which allows us to identify all the resonances within a $J-$sector at once. The blue dots correspond to the experimentally observed states. We display them both in the upper and lower plots because their spin is not determined

As mentioned, the unitarized coupled-channel model of Ref. [38], based on a $\mathrm{SU(6)}_{\mathrm{lsf}}\, \times \,$ HQSS-extended WT interaction, predicted five excited odd-parity $\Omega _c$ states with spins 1 / 2 and 3 / 2 and masses below 3 GeV (Table 1). In Fig. 1, the positions of the three $\Omega _c$ states (upper panel) and the two $\Omega _c^*$ (lower panel) are shown. We see that all masses are below 2.98 GeV, which makes difficult to identify any of them with any of the LHCb resonances. Masses and widths of other five resonances above 3 GeV are also displayed in Fig. 1. These resonances were not discussed in Ref. [38], and are much more uncertain, as they result from less attractive $\mathrm{SU(6)}_{\mathrm{lsf}} \,\times \,$ HQSS multiplets related to the exotic **4752** SU(8) irreducible representation.

All these states have been dynamically generated by solving a coupled-channel BSE using a $\mathrm{SU(6)}_{\mathrm{lsf}} \,\times \,$ HQSS-extended WT interaction as a kernel (see Sect. 2). The baryon-meson loops have been renormalized implementing one-substraction at the scale $\mu = \sqrt{\alpha \left( m_{th}^2+M_{th}^2 \right) }$, with $\alpha =1$. This RS was chosen following the works of Refs. [36, 52], where it was claimed that such a choice guarantees an approximate crossing symmetry. Moreover it also allowed for a successfully description of the $\Lambda _c(2595)$ and $\Lambda _c(2625)$ resonances, with almost^2^ no-free parameters [37].

However, it is possible to allow for some freedom and slightly modify the choice of the subtraction point by changing the value of $\alpha$. In this way, we might move up in energy the states found in Ref. [38] and compiled in Table 1, and try to identify some of them with the experimentally observed $\Omega _c^{(*)}$ states. We concentrate our study on those states as they are the ones most likely to exist since they originate from the most attractive $\mathrm{SU(6)}_{\mathrm{lsf}} \times$ HQSS representations.

**Table 2:** $\Omega _c$ and $\Omega _c^*$ states obtained using $\alpha =1.16$

Name	$M_R$ (MeV)	$\Gamma _R$ (MeV)	*J*	$M_R^{exp}$	$\Gamma _R^{exp}$
**a**	2922.2	0	1/2	–	–
**b**	2928.1	0	3/2	–	–
**c**	2941.3	0	1/2	–	–
**d**	2999.9	0.06	1/2	3000.4	4.5
**e**	3036.3	0	3/2	3050.2	0.8

**Fig. 2:**
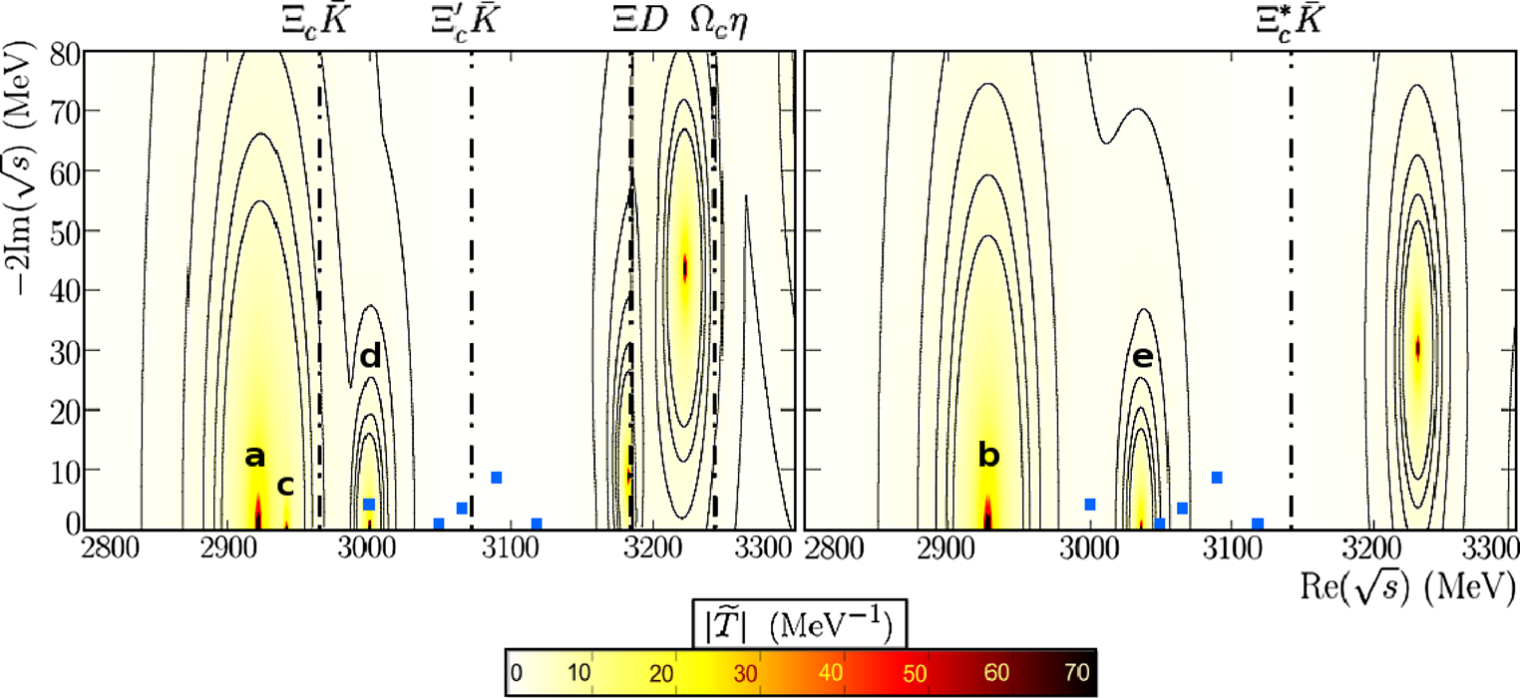
$\Omega _c$ and $\Omega _c^*$ states obtained within the scheme of Ref. [38] using $\alpha =1.16$. The left (right) plot shows the states dynamically generated for $J=\frac{1}{2}$ ($J=\frac{3}{2}$). The dotted blue points are the experimental observations, while some baryon-meson thresholds (dashed-dotted lines) are displayed for completeness. The function $|{\tilde{T}}(z)|_J$ is defined as in Fig. 1

Masses become higher when $\alpha$ becomes greater than one. Allowing for just moderately changes, we find that for $\alpha =1.16$ the two last states, labeled with **d** and **e** in Table 1, are now located near the experimental $\Omega _c(3000)$ and $\Omega _c(3050)$, with masses 2999.9 and 3036.3 MeV, respectively, while their widths are almost zero. The poles found with this new value of $\alpha$ are compiled in Table 2 and displayed in Fig. 2. Moreover, the analysis of the product of the coupling times the loop function at the pole, $g_i G_i(s_R)$, of Table 3 allows us to study the importance of the different baryon-meson channels to the dynamical generation of the $\Omega _c$ and $\Omega _c^*$ states. In particular, the state at 2999.9 MeV is mainly a $\Xi _c^{'+} {\bar{K}}$ molecular state that also couples strongly to $\Omega _c \eta$, $\Xi D$ and $\Xi _c {\bar{K}}^*$. As for the state at 3036.3 MeV, the dominant $\Xi _c^* {\bar{K}}$ channel can be reconciled with the experimentally seen decay $\Xi _c^+ K^-$, if one allows for the $\Xi _c^* {\bar{K}} \rightarrow \Xi _c {\bar{K}}$ $d-$wave transition, that does not involve the exchange of the charm-quark.

In view of the previous results, we explore a different RS to evaluate the impact of the renormalization procedure in the predictions of the $\Omega _c$ and $\Omega _c^*$ low-lying odd parity states, aiming at providing an alternative description for some of the states observed by LHCb. Thus, we allow for a variation of the subtraction constants in each channel different to that imposed within the $\mu -RS$, but still in a controlled way. For that purpose, we use the relation between the subtraction constants and the cutoff scheme given in Eqs. (13) and (14), and employ a common UV cutoff for all baryon-meson loops within reasonable limits. In this way, on the one hand, we avoid any fictitious reduction of any baryon-meson channel by using a small value of the cutoff and, on the other hand, we prevent an arbitrary variation of the subtraction constants,^3^since we correlate all of them to a reasonable value of the UV cutoff, while still keeping the full analyticity of the baryon-meson loops, as discussed below Eq. (14).

**Table 3:** Properties of the $\Omega _c(2999.9)$ and $\Omega _c^*(3036.3)$ states, labeled as poles **d** and **e**, respectively, obtained using $\alpha =1.16$. The first column displays the different baryon-meson channels coupled to $\Omega _c(2999.9)$, ordered by their threshold energies, in the $J=1/2$ sector. The second and third columns show the absolute value of the coupling and the product of the coupling times the loop function at the pole position, respectively, for all baryon-meson coupled states. The fourth, fifth and sixth columns are equivalent to the first three columns but for $\Omega _c^*(3036.3)$ in the $J=3/2$ sector

$J=1/2$	pole **d**		$J=3/2$	pole **e**	
Channel	|*g*|	$gG {\text { (MeV)}}$	Channel	$\ |g|$	$gG {\text { (MeV)}}$
$\Xi _c \bar{K}$	0.1	$-\,1.4 + 0.3 j$	$\Xi ^*_c \bar{K}$	1.9	$-\,26.6 -\,0.1 j$
$\Xi _c' \bar{K}$	1.8	$-\,27.1$	$\Omega _c^* \eta$	1.7	16.3
$\Xi D$	1.7	10.4	$\Xi D^*$	1.6	$-\,8.5$
$\Omega _c \eta$	1.7	15.7	$\Xi _c \bar{K}^*$	1.6	$-\,14$
$\Xi D^*$	0.8	$-\,3.5 -\,0.1 j$	$\Xi ^* D$	0.5	$-\,2.7$
$\Xi _c \bar{K}^*$	1.3	10.1	$\Xi '_c \bar{K}^*$	0.6	$-\,4.9$
$\Xi '_c \bar{K}^*$	1.1	$-\,7.3 -\,0.2 j$	$\Omega _c \omega$	0	0.3
$\Omega _c \omega$	0.1	0.7	$\Xi ^*_c \bar{K}^*$	1.3	$-\,8.9$
$\Xi ^*_c \bar{K}^*$	0.6	$3.6 -\,0.2 j$	$\Xi ^* D^*$	0.6	$-\,2.4$
$\Xi ^* D^*$	0.7	$-\,2.6$	$\Omega _c^* \omega$	0.1	0.4
$\Omega _c^* \omega$	0	0	$\Omega D_s$	0.8	$-\,3.3$
$\Omega _c \eta '$	0.5	2.5	$\Omega _c \phi$	0.6	3.5
$\Omega _c \phi$	1.1	$5.4 + 0.1 j$	$\Omega _c^* \eta '$	0.5	2.8
$\Omega D_s^*$	1.2	$-\,3.7$	$\Omega D_s^*$	1	$-\,3.4$
$\Omega _c^* \phi$	0.6	$-\,2.9 + 0.1 j$	$\Omega _c^* \phi$	1.2	6.5

**Table 4:** $\Omega _c$ and $\Omega _c^*$ states calculated using the subtraction constants associated to a cutoff of $\Lambda =1090$ MeV. We identify experimentally two $J=1/2$ and one $J=3/2$ states

Name	$M_R$ (MeV)	$\Gamma _R$ (MeV)	*J*	$M_R^{exp}$	$\Gamma _R^{exp}$
**a**	2963.95	0.0	1/2	–	–
**c**	2994.26	1.85	1/2	3000.4	4.5
**b**	3048.7	0.0	3/2	3050.2	0.8
**d**	3116.81	3.72	1/2	3119.1/ 3090.2	1.1/ 8.7
**e**	3155.37	0.17	3/2	–	–

**Table 5:** $J=1/2$ $\Omega _c$ states, labeled as poles **a**, **c** and **d**, calculated using the subtraction constants determined by a unique UV cutoff $\Lambda =1090$ MeV (see Eq. (13)). The first column displays the different baryon-meson coupled channels, ordered by their threshold energies. The subsequent columns show the absolute value of the coupling and the product of the coupling times the loop function at the pole for all baryon-meson coupled states for pole **a** at 2963.95 MeV (second and third columns), pole **c** at 2994.26 MeV (fourth and fifth columns) and pole **d** at 3116.81 MeV (sixth and seventh columns). Poles **c** at 2994.26 MeV and **d** at 3116.81 MeV might be identified with the experimental $\Omega _c(3000)$ and the $\Omega _c(3119)$ or $\Omega _c(3090)$, respectively

Channel	Pole **a**		Pole **c**		Pole **d**	
	|*g*|	$gG \text {(MeV)}$	|*g*|	$gG \text {(MeV)}$	|*g*|	$gG \text {(MeV)}$
$\Xi _c \bar{K}$	0.9	$-\,33.0 -\,0.1 j$	0.3	$-\,10.2 +\, 6.0 j$	0.3	$-\,11.7 +\, 2.2 j$
$\Xi _c' \bar{K}$	0.4	$-\,7.3$	1.7	$39.1 +\, 0.9 j$	0.0	$-\,0.6 +\, 0.1 j$
$\Xi D$	1.8	10.1	1.0	$-\,6.4 -\,2.1 j$	2.3	$-\,26.9 -\,1.1 j$
$\Omega _c \eta$	0.4	4.1	1.9	$-\,22.7 -\,0.5 j$	0.3	$-\,4.6$
$\Xi D^*$	1.7	3.6	1.4	$3.5 -\,0.9 j$	2.2	$12.5 -\,0.8 j$
$\Xi _c \bar{K}^*$	0.0	$-\,0.1$	1.8	$-\,8.7 + \,0.2 j$	1.8	$17.4 + \,0.1 j$
$\Xi '_c \bar{K}^*$	0.9	0.4	1.4	$1.8 -\,0.3 j$	0.2	$-\,0.7 -\,0.6 j$
$\Omega _c \omega$	0.5	$-\,0.4$	0.6	$-\,1.0 +\, 0.2 j$	0.3	$1.7 + \,0.1 j$
$\Xi ^*_c \bar{K}^*$	1.2	$-\,2.0$	0.3	$0.1 + \,0.2 j$	1.5	$3.8 -\,0.4 j$
$\Xi ^* D^*$	0.2	0.4	0.9	$1.7 -\,0.1 j$	2.5	$0.4 -\,0.1 j$
$\Omega _c^* \omega$	0.4	0.5	0.1	0.0	0.9	$-\,2.7 +\, 0.1 j$
$\Omega _c \eta '$	0.1	$-\,0.6$	0.2	$1.0 +\, 0.1 j$	0.6	0.8
$\Omega _c \phi$	0.4	2.6	1.1	$7.2 -\,0.6 j$	0.1	$0.2 -\,0.3 j$
$\Omega D_s^*$	0.3	2.0	0.1	$-\,0.8 -\,0.4 j$	1.9	$-\,9.2 -\,0.2 j$
$\Omega _c^* \phi$	0.8	6.5	0.4	$-\,2.8 -\,1.2 j$	0.6	$3.4 -\,0.5 j$

To identify our five dynamically generated $\Omega _c$ and $\Omega _c^*$ states of Table 1 using the new subtraction constants, we first need to determine how the masses (and widths) of our generated states change as we adiabatically vary the values of the subtraction constants. This can be done by

19 $$\begin{aligned} G_i(s) = \overline{G}_i(s)-(1-x) \overline{G}_i(\mu ^2)+x G_i^{\Lambda }(s_{i+}), \end{aligned}$$

where *x* is a parameter that changes slowly from 0 to 1, and $\mu ^2=(m_{th}^2+M_{th}^2)$. In this manner, we can follow in the complex energy plane the original $\Omega _c$ and $\Omega _c^*$ as we modified our prescription to use a common cutoff for the computation of the subtraction constants.

Our results for the $\Omega _c$ and $\Omega _c^*$ are shown in Table 4 for a fixed cutoff of $\Lambda =1090$ MeV. In this case, we find that three poles (those previously named **c**, **b** and **d**) can be identified with the three experimental states at 3000, 3050 and 3119 or 3090 MeV. The identification is possible not only due to the closeness in energy to the experimental ones but also because of the dominant contribution of the experimental $\Xi _c {\bar{K}}$ and $\Xi _c^{'} {\bar{K}}$ channels to their dynamical generation. The contribution is measured by the product *gG* at the pole, as reported in Table 5 for $J=1/2$ and Table 6 for $J=3/2$. For the $J=1/2$ state at 2994 MeV (pole **c**), we observe a significant contribution of the $\Xi _c^{'} {\bar{K}}$ and $\Xi _c {\bar{K}}$ channels, while $\Omega _c \eta$ is also relevant. We identify this state with $\Omega _c(3000)$. As for the $J=1/2$ state at 3117 MeV (pole **d**), the dominant contribution comes from $\Xi D$ but also from $\Xi _c {\bar{K}}^*$, $\Xi D^*$ and $\Xi _c {\bar{K}}$. Thus, we can identify this state with $\Omega _c(3119)$ or the $\Omega _c(3090)$ given its proximity in mass. Moreover, a sizable width of $8.7 \pm 1.0 \pm 0.8$ MeV is reported for the latter state in Ref. [1] to be compared with the one around 4 MeV found here for the state **d**. Finally, the $J=3/2$ state at 3049 MeV (pole **b**) could be identified with $\Omega _c (3050)$ as it couples strongly to $\Xi _c^* {\bar{K}}$ and $\Xi _c{\bar{K}}^*$, channels connected to $\Xi _c {\bar{K}}$ by $d-$wave transitions, while having also an important contribution from $\Omega _c^* \eta$. In summary, two $J=1/2$ and one $J=3/2$ can be identified experimentally for a cutoff of $\Lambda =1090$ MeV.

**Table 6:** $J=3/2$ $\Omega _c^*$ states, labeled as poles **b** and **e**, calculated using the subtraction constants determined by a unique UV cutoff $\Lambda =1090$ MeV (see Eq. (13)). The first column displays the different baryon-meson coupled channels, ordered by their threshold energies, for $J=3/2$. The subsequent columns show the absolute value of the coupling and the product of the coupling with the loop function at the pole for all baryon-meson coupled states for pole **b** at 3048.7 MeV (second and third columns) and pole **e** at 3155.37 MeV (fourth and fifth columns). Pole **b** at 3048.7 MeV might be identified with the experimental $\Omega _c(3050)$

Channel	Pole **b**		Pole **e**	
	|*g*|	$gG \text {(MeV)}$	|*g*|	$gG \text {(MeV)}$
$\Xi ^*_c \bar{K}$	1.8	$-\,38.8 -\,0.1 j$	0.1	$-\,4.3 + 0.1 j$
$\Omega _c^* \eta$	1.8	20.1	0.8	$13.3 -\,0.3 j$
$\Xi D^*$	0.8	$-\,3.0$	3.6	$-\,24.4$
$\Xi _c \bar{K}^*$	2.1	$-\,14.0$	0.9	$10.5 + 0.2 j$
$\Xi ^* D$	0.9	1.9	2.2	$-\,10.7$
$\Xi '_c \bar{K}^*$	0.5	$-\,1.3$	0.1	$-\,0.6 + 0.1 j$
$\Omega _c \omega$	0.3	1.0	0.4	$-\,2.9$
$\Xi ^*_c \bar{K}^*$	1.2	$-\,0.7$	0.6	$2.4 + 0.1 j$
$\Xi ^* D^*$	1.1	$-\,1.2$	2.4	$-\,2.3$
$\Omega _c^* \omega$	0.4	0.4	0.2	$-\,1.0$
$\Omega D_s$	0.1	$-\,0.4$	1.4	2.1
$\Omega _c \phi$	0.5	$-\,2.6$	0.2	$-\,0.4$
$\Omega _c^* \eta '$	0.1	$-\,0.5$	0.8	$-\,2.0$
$\Omega D_s^*$	0.2	$-\,1.1$	1.9	8.1
$\Omega _c^* \phi$	1.1	$-\,7.6$	0.1	0.4

**Fig. 3:**
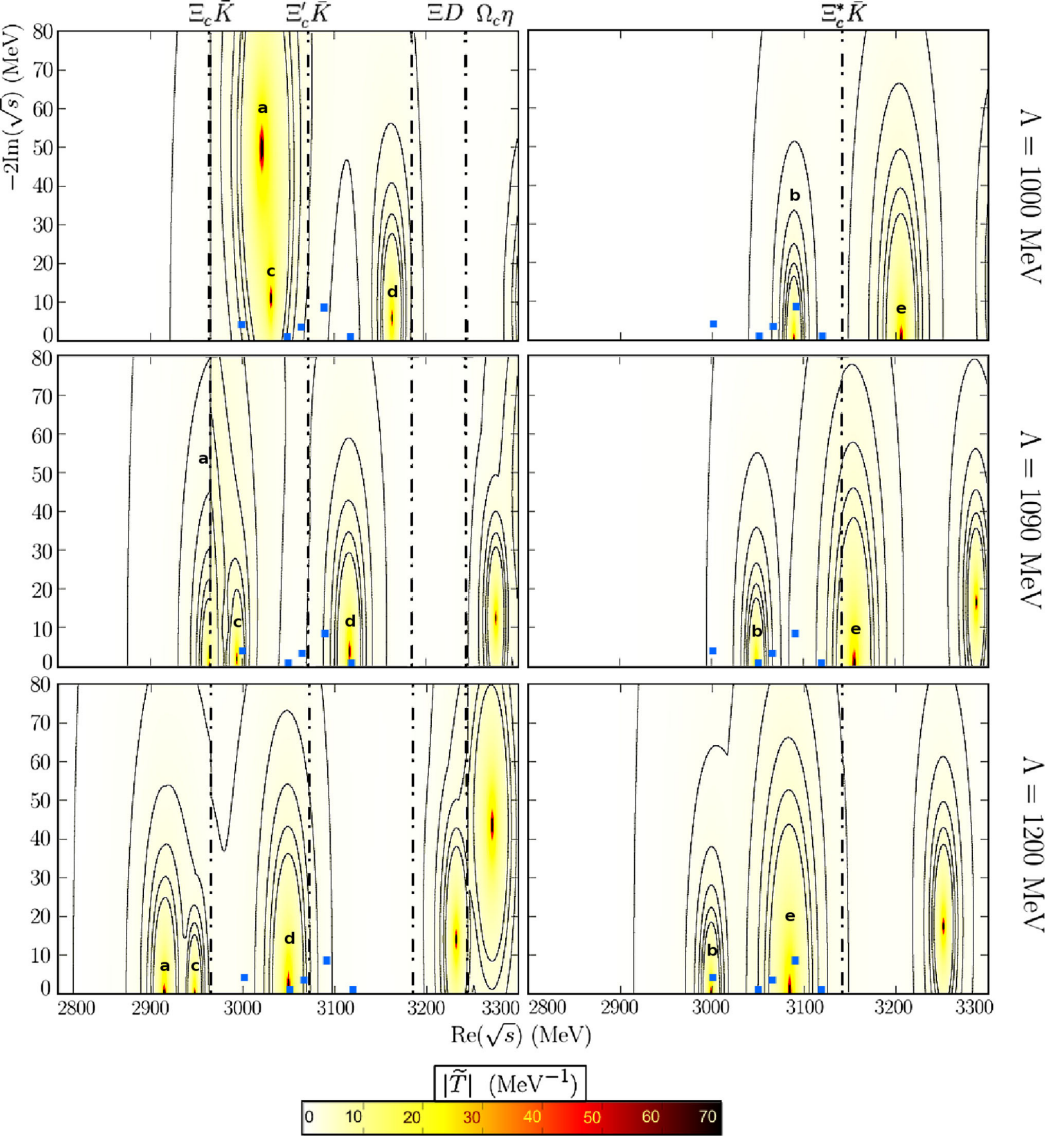
$\Omega _c$ and $\Omega _c^*$ states for different UV cutoffs. The blue squares indicate the experimental points. Dashed-dotted lines represent the closest baryon-meson thresholds. The left plots are for $J=\frac{1}{2}$ and the right ones for $J=\frac{3}{2}$, while the function $|{\tilde{T}}(z)|_J$ is defined as in Fig. 1. For the two largest values of $\Lambda$, some resonant states from less attractive $\mathrm{SU(6)}_{\mathrm{lsf}}\times$ HQSS multiplets, stemming from the exotic **4752** SU(8) representation, are also visible in the region of higher masses

In order to assess the dependence of our results on the cutoff, we have examined lower and higher values. As indicated before, the variation in the cutoff scale changes the value of the subtraction constant. This variation is related to the change of the size of higher order corrections in the meson-baryon scattering amplitude that are not known and not fixed by unitarization. Below 800 MeV, all resonances become heavier and much wider than the observed LHCb states. Actually, a clear identification between our results and some of the experimental states is not possible until a value of $\Lambda \sim 1000$ MeV. For cutoffs bigger than 1300–1350 MeV, the $\Omega _c$ and $\Omega _c^*$ states coming from the most attractive $\mathrm{SU(6)}_{\mathrm{lsf}} \times$ HQSS representations appear well below 3 GeV, and we can neither make an identification between those states and the LHCb spectrum. In Fig. 3, we show the obtained pole positions for $\Lambda = 1090$ MeV (Table 4) and two additional cutoffs, around 100 MeV smaller and bigger, respectively, than this central one. It can be seen that for $\Lambda =$1090 and $\Lambda =$1200 MeV, a maximum number of three states can be identified. As compared to the $\Lambda =$1090 MeV case previously discussed, for $\Lambda =$1200 MeV we can identify two $\Omega _c^*$ states with $J=3/2$ at 3000 and 3090 MeV, whereas a $J=1/2$ $\Omega _c$ is seen at 3050 MeV. The $J=1/2$ state at 3050 MeV corresponds now to the **d** state, that for $\Lambda =1090$ MeV was identified with the $\Omega _c(3119)$ or $\Omega _c(3090)$ resonances, and it has a dominant $\Xi D$ component. It might still be the $\Omega _c(3090)$. The $J=1/2$ **c** pole now moves well below 3 GeV and this makes difficult its identification with any of the LHCb states. In the $J=3/2$ sector, the resonance that appears a 3000 MeV is the pole **b** and strongly couples to $\Xi _c^* {\bar{K}}$ and $\Xi _c{\bar{K}}^*$, as already mentioned above. The additional $J=3/2$ state at 3090 MeV is the pole **e** in the nomenclature used in Table 4 for $\Lambda =1090$ MeV, and as it can be seen there, it has a large $\Xi D^*$ molecular component, and it could be associated to the $\Omega _c(3119)$ or $\Omega _c(3090)$ LHCb resonances. In all three cases and in order to make the experimental identification possible, a significant coupling to the $\Xi _c {\bar{K}}$ channel could be obtained, often via $\Xi _c^* {\bar{K}}$ and $\Xi _c {\bar{K}}^*$ allowing for the $d-$wave transitions. In summary we see that by changing the UV cutoff, the pole positions of the dynamically generated states are modified making more plausible different identifications between some of these states and those observed by LHCb.

As mentioned in the Introduction, the molecular nature of the five $\Omega _c$ narrow states has been recently analyzed in Refs. [39, 40] as well as the observed broad structure around 3188 MeV in Ref. [55]. In Ref. [39] the interaction of the low-lying mesons (pseudoscalar and vector mesons separately) with the ground-state $1/2^+$ baryons in the $C=+1$, $S-\,2$ and $I=0$ sector has been built from *t*-channel vector meson exchanges. Two $J=1/2$ baryon-meson molecular states could be identified with the experimental $\Omega _c(3050)$ and $\Omega _c(3090)$, mostly having the state at 3050 MeV a $\Xi _c^{'} {\bar{K}}$ component with an admixture of $\Omega _c \eta$, while the 3090 MeV would be a $\Xi D$ molecule. These results have been reproduced in the $J=1/2$ sector in Ref. [40], within a local hidden gauge approach extended to the charm sector that also incorporates baryon $3/2^+$-pseudoscalar meson components. This is because the diagonal terms in the interaction kernel are the same in both models and these two $\Omega _c$ states do not couple to baryon $1/2^+$-vector meson channels in Refs. [39, 40]. Furthermore, by incorporating baryon $3/2^+$-pseudoscalar meson states, a $J=3/2$ baryon-meson molecular state has been also identified in Ref. [40] with the experimental $\Omega _c(3119)$. This state would be a baryon $3/2^+$- pseudoscalar meson molecule with large couplings to ${\bar{K}} \Xi _c^*$ and $\Omega ^*_c\eta$.

In this work and for $\Lambda =1090$ MeV, we have also obtained three baryon-meson molecular states that couple predominantly to ${\bar{K}} \Xi _c^{'}$, $D \Xi$ and ${\bar{K}} \Xi _c^*$, respectively, but with a different experimental assignment of masses, that is, $J=1/2$ $\Omega _c(3000)$ and $J=1/2$ $\Omega _c(3119)$ or $\Omega _c(3090)$, and $J=3/2$ $\Omega _c(3050)$, which correspond to poles **c** and **d**, and **b**, respectively. However, the $g_i G_i(s_R)$ strengths for the dominant channels found in this work are in reasonable good agreement with those given in Ref. [40]. As we have illustrated in Fig.  3, our predictions for masses are subjected to sizeable uncertainties, which might lead to confusions in the assignments to the LHCb states proposed in this work.

Nevertheless we should highlight that, we use here a different regularization scheme of the loop functions and different interaction matrices than in the works of Refs. [39, 40] that should explain the differences found. Note that the matrix elements involving the interaction of Goldstone-bosons and heavy-baryons are fixed by chiral symmetry and should agree in the three approaches. The differences come from channels involving *D*, $D^*$ and light-vector mesons, where HQSS does not completely fix the interactions. Furthermore, in the models of Refs. [39, 40] some HQSS breaking terms suppressed by the heavy-quark-mass are accepted. In addition, we incorporate the mixing of channels involving pseudoscalar mesons with channels involving vector mesons, while such mixings are claimed to be negligible in the case of Ref. [40]. Our model also incorporates the contribution of baryon-meson states of higher mass than those included in Refs. [39, 40], though, those heavier baryon-meson channels do not give any relevant contribution to the generation of the low-lying $\Omega _c$ and $\Omega _c^*$ states.

In Ref. [55] the broad structure observed by the LHCb Collaboration around 3188 MeV has been analysed as the superposition of two $D \Xi$ bound states within the Bethe–Salpeter formalism in the ladder and instantaneous approximation. As can be seen in Fig. 3. we also generate resonances in this region, but it is difficult to reach any conclusion since most likely, we would have to consider also some states from less attractive $\mathrm{SU(6)}_{\mathrm{lsf}}\, \times \,$ HQSS multiplets, stemming from the exotic **4752** SU(8) representation [38]. A candidate of a loosely bound molecular state with a large $\Xi ^*_c{\bar{K}}$ component and a mass around 3140 MeV is also predicted in Ref. [56]. It results from $\Xi ^*_c{\bar{K}}/\Xi _c{\bar{K}}^*/\Xi _c^\prime {\bar{K}}^*$ coupled-channel dynamics using a one-boson-exchange potential. It is difficult to associate such state with any of the predictions obtained here from the scheme of Ref. [38], since the work of Ref. [56] does not consider $\Xi ^{(*)}D^{(*)}$ channels.

## Conclusions

We have reviewed the RS used in the unitarized coupled-channel model of Ref. [38] and its impact in the $C=1$, $S=- \,2$, and $I=0$ sector, where five $\Omega _c$ states have been recently observed by the LHCb Collaboration [1]. A coupled-channel BSE, with a $\mathrm{SU(6)}_{\mathrm{lsf}} \,\times \,$ HQSS-extended WT meson-baryon interaction, is solved in [38] within the on–shell approximation, and adopting a one-subtraction RS at fixed scale for all channels, as advocated in Refs. [36, 52]. Five odd-parity $\Omega _c, \Omega ^*_c$ states, coming from the most attractive $\mathrm{SU(6)}_{\mathrm{lsf}} \,\times \,$ HQSS representations, are dynamically generated, but with masses below 2.98 GeV that cannot be easily identified with any of the LHCb resonances, located all of them above 3 GeV. Predicted masses can be moved up by implementing a different RS. We have explored two different scenarios, introducing at most only one additional undetermined parameter in the scheme. In the first one, the common energy-scale used in [38] to perform the subtractions is modified allowing for moderate variations. In the second one, a common UV cutoff is used to render finite the UV divergent loop functions in all channels. In both cases, we could move two or three states in the region between 3 and 3.1 GeV, where the LHCb resonances lie. In particular, when we use $\Lambda =1090$ MeV, we obtain three baryon-meson molecular states (poles **c** and **d**, and **b**) that couple predominantly to ${\bar{K}} \Xi _c^{'}$, $D \Xi$ and ${\bar{K}} \Xi _c^*$, and can be easily related to the LHCb resonances and to results of Refs. [39, 40]. Thus for the dominant channels, we obtain strengths for the wave function at the origin in a reasonable good agreement with those found in Ref. [40]. There exist, however, some disagreements in the predictions for the masses, which need to be taken with some caution. At least, our predictions for masses are subjected to sizable uncertainties, which might lead also to confusions in the assignments to the LHCb states proposed in this work.

In summary, we can conclude that some (probably at least three) of the states observed by LHCb [1] will have odd parity and spins $J=1/2$ and $J=3/2$. Moreover, those associated to the poles **b** with $J=3/2$ and **c** with $J=1/2$ would belong to the same $\mathrm{SU(6)}_{\mathrm{lsf}}$ $\times$ HQSS multiplets [38, 42] that the strangeness-less $\Lambda _c(2595)$ and $\Lambda _c(2625)$, and $\Lambda _b(5912)$ and $\Lambda _b(5920)$ resonances in the charm and bottom sectors, respectively.

## Footnotes

1 Often we refer to all poles generically as resonances, regardless of their concrete nature, since usually they can decay through other channels not included in the model space.

2 Only, the $\alpha$ parameter in Eq. (12) was slightly reduced from the default value of 1 advocated in Refs. [36, 52].

3 This will induce an enormous freedom difficult to fix with the scarce available data.
